# Application of ChatGPT in reducing vaccine hesitancy and enhancing vaccine acceptance: hope or myth?

**DOI:** 10.1590/1806-9282.20231558

**Published:** 2024-05-20

**Affiliations:** 1 Graphic Era Deemed to be University, Department of Biotechnology – Dehradun, Uttarakhand, India.

Vaccines have always remained the ultimate scope of health recovery from many diseases. In recent years, with the emerging COVID-19 pandemic, several vaccines have been approved by the regulatory agencies of various countries and were administered in different forms^1^. However, before administration of vaccines to the affected ones, several human lives ended up due to lack of vaccines and hesitancy of taking vaccines, which made them step back from taking vaccines^2^. This condition was described as "vaccine hesitancy." World Health Organization (WHO) defined this condition as a state of mind to "the delay in the acceptance or refusal to vaccinate despite the availability of vaccine services^3^." Vaccine hesitancy is reserved not only for a single person but also for several communities due to the spread of misinformation as well as misunderstanding about the vaccines among public. However, it was difficult to control the spread of misinformation. With the help of social media, the benefits of vaccination have been rapidly spread to public by giving examples through case observations that achieved success and gained back their normal health^4,5^. Recently, with the introduction of ChatGPT, which is a text-generative artificial intelligence (AI) tool, awareness has been spread in favor of vaccine acceptance, which has helped several people to assess the benefit–risk of vaccines and encouraged users to get vaccinated and reduce misconceptions^2^. Therefore, it has become essential to study the application of ChatGPT in reducing vaccine hesitancy to enhance vaccine acceptance.

The achievement of this era is the application of AI with the launch of ChatGPT. This supportive online tool was introduced during the COVID-19 pandemic^6^. Evidence-based studies have shown that public’s trust on such tools regarding the COVID-19 vaccination has created a range of hesitancy, and only a few people were willing to get vaccinated^7-9^. Recently, a study was performed on COVID-19 vaccination in Cyprus, which reveals that two-thirds of medical practitioners have opposed mandatory COVID-19 vaccination^10^, and a similar response was obtained from French hospital workers^11^. This is still a myth in some communities and a challenge for public health professionals due to resistance against vaccination. Recently, common people and health professionals have set a list of questions to be answered by ChatGPT on vaccine hesitancy and found that the responses provided were clear, correct, and concise^12^. Most studies that were correlated and retrieved from ChatGPT reveal that misinformation regarding the COVID-19 vaccines is likely to avoid preventive behaviors, propagating vaccine hesitancy and negative attitudes toward the COVID-19 vaccines^13,14^.

Apart from providing accurate information on vaccine hesitancy, surprisingly, ChatGPT also undergoes technical and ethical issues, which are not a substitute for the findings of a scientific, or medical expert. Technically, it can match the information tuned toward information that aligns well with scientific evidence, but practically, one cannot solely rely on it for decisions related to medical practice. In some instances, inaccurate information may be generated depending on variations in the versions of the ChatGPT^15^. So, displaying inappropriate content is possible with AI tools, leading to confusion among the public. There is a possibility that ChatGPT may provide limited information due to a lack of understanding of the globe and events after 2021 and may end up replying, "My knowledge cutoff is 2021." ChatGPT lacks practical patient care options that differ according to ethnicity and medical troubleshooting. Information provided by ChatGPT lacks a descriptive nature, which lacks a match with the quantitative statistical analysis, thereby creating a lag in the technical aspect^16^. Although ChatGPT promisingly provides users with vaccine hesitancy information, it is critical to acknowledge its limits and, when needed, primarily, its availability is neutral when willing to make vaccine acceptance decision. However, for the public with limited medical knowledge, without the consultation of an expert’s medical advice, these tools are not accessible from the risk of eliciting misleading responses^12^.

Furthermore, in the future, the implementation of ChatGPT in helping the public get vaccinated for COVID-19 becomes a significant medical application in discussing the concerns about immune-based variations globally. Through programming the tool for computer-aided diagnosis, clinicians can provide case studies as reference studies for further investigation. They can check the match line data provided by ChatGPT with clinical variables. Therefore, the healthcare ecosystem is realizing to balance the clinical condition of patients by seeking ChatGPT in the next-generation healthcare technology. It is believed that ChatGPT can bring improvements to any process within healthcare operation and delivery^2^.

Overall, fake and irrelevant questions related to vaccines and vaccination protocol can be captured by ChatGPT because the language applied by this AI is not overly technical, and one can understand and make better decision toward vaccine acceptance. Moreover, fearless, supportive opinions are helpful for the public in changing their perceptions about taking vaccines, encouraging users to get vaccinated, and reducing misconceptions.
